# Small individual loans and mental health: a randomized controlled trial among South African adults

**DOI:** 10.1186/1471-2458-8-409

**Published:** 2008-12-16

**Authors:** Lia CH Fernald, Rita Hamad, Dean Karlan, Emily J Ozer, Jonathan Zinman

**Affiliations:** 1 School of Public Health, University of California, Berkeley, USA; 2 Yale University, New Haven, USA; 3 Innovations for Poverty Action, New Haven, USA; 4 Jameel Poverty Action Lab, Massachusetts Institute of Technology, Cambridge, USA; 5 Dartmouth College, Hanover, USA

## Abstract

**Background:**

In the developing world, access to small, individual loans has been variously hailed as a poverty-alleviation tool – in the context of "microcredit" – but has also been criticized as "usury" and harmful to vulnerable borrowers. Prior studies have assessed effects of access to credit on traditional economic outcomes for poor borrowers, but effects on mental health have been largely ignored.

**Methods:**

Applicants who had previously been rejected (n = 257) for a loan (200% annual percentage rate – APR) from a lender in South Africa were randomly assigned to a "second-look" that encouraged loan officers to approve their applications. This randomized encouragement resulted in 53% of applicants receiving a loan they otherwise would not have received. All subjects were assessed 6–12 months later with questions about demographics, socio-economic status, and two indicators of mental health: the Center for Epidemiologic Studies – Depression Scale (CES-D) and Cohen's Perceived Stress scale. Intent-to-treat analyses were calculated using multinomial probit regressions.

**Results:**

Randomization into receiving a "second look" for access to credit increased perceived stress in the combined sample of women and men; the findings were stronger among men. Credit access was associated with reduced depressive symptoms in men, but not women.

**Conclusion:**

Our findings suggest that a mechanism used to reduce the economic stress of extremely poor individuals can have mixed effects on their experiences of psychological stress and depressive symptomatology. Our data support the notion that mental health should be included as a measure of success (or failure) when examining potential tools for poverty alleviation. Further longitudinal research is needed in South Africa and other settings to understand how borrowing at high interest rates affects gender roles and daily life activities. CCT: ISRCTN 10734925

## Background

### Poverty and mental health

Over 300 million people in Africa live in abject poverty, surviving on less than $1 per day [1]. In South Africa, about 4.8 million people live in poverty, which represents 10.7% of the population. Although a portion of the population is very wealthy, conditions are still adverse for much of the population. Living with low socioeconomic status (SES) is a root cause of poor health, and contributes to reduced life expectancy, increased rate of disease, and lower perceived quality of life, both in developing and developed countries [2-8]. These well-known associations between low SES and poor physical health have been extended to mental health as well. A large body of literature from the developing [9-12] and developed world [13-19] supports the idea that low SES is also a risk factor for worse mental health outcomes, including measures of psychological distress, depression, anxiety and other disorders.

### Microcredit and small individual loans

Microcredit programs (e.g., the Grameen Bank, other non-governmental organizations (NGO's), and government lending programs) – which provide small loans to individuals who are ineligible for traditional and potentially cheaper financial services – have been hailed as one solution to improving conditions for people living in poverty [20]. The assumption behind embedding microcredit loans into other social interventions is that clients will use the income to invest in small businesses in order to support their families and children. Microcredit programs have been described as a tool for "large population groups to find ways to break out of poverty" [21]. Clients typically form groups with other members of their community and receive the loans jointly, thereby providing social collateral to substitute for their lack of physical collateral. Although microcredit programs were first initiated about three decades ago in South Asia and Latin America, there are currently hundreds of programs in sub-Saharan Africa, a testament to microcredit's perceived potential for social and economic development [22].

The "cash loan" industry – another source of small loans for people living in poverty – has important differences from and similarities to "traditional microcredit". Most microcredit loans are delivered by lenders with explicit social welfare and targeting goals; micro-lenders typically target female entrepreneurs and often use group liability mechanisms. However, the industrial organization of microcredit is trending steadily in the direction of the for-profit, more competitive delivery of individual liability credit, often without targeting to entrepreneurs and instead to employed individuals (often referred to as "cash loan" industry) [23,24]. This change is happening both from the bottom-up (non-profits converting to for-profits) as well as from the top-down (for-profits expanding into microcredit segments), and represents the future for many traditional microcredit interventions.

Similar to microcredit borrowers, cash loan borrowers typically lack the credit rating and/or collateralizable wealth needed to borrow from traditional institutional sources such as commercial banks. Cash loan sizes tend to be small relative to the fixed costs of underwriting and monitoring them, but substantial relative to borrower income. For example, the median loan size made in this experiment ($127) was 40% of the median borrower's gross monthly income (6.31 Rand = $1). The loan providers compete in an industry segment that offers small, high-interest, short-term, uncollateralized credit with fixed repayment schedules to a "working poor" population.

### Access to credit and related health outcomes

A review of the available observational evidence suggests that microcredit programs may improve the economic conditions of clients in a variety of settings based on indicators such as savings, income, and assets [25]. Beyond economic benefits, there has been a great deal of interest in microcredit and the growth of expensive/subprime credit markets that provide small cash loans as means for improving lives of participants in social and health domains as well [26].

Increased income has been conceptualized as the primary pathway through which microcredit could improve health outcomes, both physical and mental [27]. First, additional income to the family could allow households to purchase more or better quality food, medicines when necessary, or to add structural improvements to their homes, all of which could positively influence health and reduce stressors. The increased income could allow participants to invest in income-generating activities – such as those promoted in traditional microcredit programs – and these could alleviate stress relating to sources of future income. Importantly, access to credit has been emphasized as a promising strategy for poor women in particular to increase their control of economic resources and decision-making power, potentially enhancing self-esteem and decreasing perceived stress and depression [27,28]. On the other hand, incurring loan debt could certainly increase financial strain and psychological stress for some poor families, especially if they struggle with repayment. Having to cope with debt has been associated with poor mental health in some other studies conducted in high-income countries [15,29,30].

There is a small but growing empirical literature on how microcredit loans are utilized and the extent to which recipients – particularly women in developing countries – experience the hypothesized benefits in "agency" such as greater economic independence, decision-making power, and reductions in domestic violence. This literature – a combination of qualitative, cross-sectional, and non-experimental longitudinal evaluations – has shown mixed evidence of success. For example, a study of a combined micro-credit and participatory research intervention in South African villages that used a longitudinal, randomized design found evidence for reductions in physical and sexual violence [31,32]. Using qualitative and quantitative analyses, the authors also found evidence of higher levels of structural social capital (e.g. social networks) and cognitive social capital (e.g. perceptions of solidarity and reciprocity) in the intervention group [33].

Prior studies of micro-credit loan programs in other cultural contexts, however, have noted that women's participation has led to only modest benefits or, in some cases greater marital conflict and violence [34]. A case-control study among Bangladeshi women comparing those who participated in microcredit with those didn't showed only small improvements in some domains of decision-making power among clients [35], one of the proposed pathways towards empowerment and improved mental health [36]. These changes occurred primarily in domains where women already held sway, such as food and education purchases, suggesting that microcredit's failure to address broader social norms in the specific cultural context was responsible for the maintenance of the status quo. Similarly, survey research [35] in South India suggested that the effects of microcredit on women's decision-making power in highly patriarchal contexts is limited in the absence of additional program components that help to shift traditional gender-based norms [37]. A study involving in-depth case studies with 20 rural Nigerian women noted the range of obstacles (e.g. spousal control, the government, and geographic distance) that women had to confront in paying back their microcredit loans [38].

The conflicting pattern of results found thus far in the microcredit literature on empowerment is likely to be attributable to the heterogeneity of approaches and components among the programs studied, divergent methods used for defining and assessing key outcomes, distinctive cultural contexts in which programs are evaluated, and variability of women's experiences even within the same intervention [37,39,40].

### Microcredit and mental health

The present study focuses specifically on the effects of microcredit on levels of perceived stress and psychological functioning of participants, dimensions that have received little research attention thus far. To our knowledge, only two published studies have systematically examined the association between participation in microcredit and psychological functioning. A large cross-sectional study of low-income women conducted in South India found that being a member of a microcredit intervention ("self help group") for greater than two years was associated with lower levels of self-reported emotional stress when compared with not being part of the program at all; there was no significant association between emotional stress and being a member of the intervention for less than two years, suggesting a potential duration effect of the microcredit program [40]. Another cross-sectional study conducted in Bangladesh compared poor women who had participated in a microcredit program with those who had not. No differences were found in self-reported emotional stress; although women who had participated in microcredit reported lower levels of social withdrawal in response to stressful events, and they also reported more fatalistic attitudes [41]. The authors attributed the lack of clear psychological benefits for the women who had participated in microcredit to a "discrepancy between expectation and achievement". The authors postulated that the "anxieties and tensions from newly adopted nontraditional roles" were adversely affecting women's emotional wellbeing.

The small quantitative literature on the psychological impact of microcredit interventions is limited by lack of random assignment to Treatment or Control; thus selection bias and survivorship bias unavoidably influence the ability to make clear attributions [42-44]. The study reported here addresses this critical research gap by presenting data from a randomized controlled evaluation of consumer credit access in the cash loan market that serves low-income working adults in South Africa. As discussed in detail below, the intervention tested here differs from some of the other community-based approaches to microcredit in the literature that combine loans with other kinds of social programs or community organizing efforts.

### Aims and hypotheses of current study

Given the potentially important impact of microcredit programs on mental health outcomes, we investigated the effects of participating in a small, individual cash loan program on depressive symptoms and perceived stress in a sample of adult women and men. We have shown previously that adults in this population exhibited high numbers of symptoms of poor mental health, and also that these outcomes were worse for those living with low SES [10]. As noted earlier, the small extant literature that examines the relationship between microcredit participation and social power for women has found mixed evidence for benefits.

In the present study, we hypothesized that improved access to a credit program and consequent improvements in SES would be associated with positive impacts on mental health in this population due to the alleviation of stress related to poverty. Our expectations regarding positive effects, however, were tempered by the recognition of the alternative possibility that the introduction of debt and burden of repayment could cause an increase in stress and depressive symptoms.

Prior research on the contextual barriers to women's take-up of microcredit cited earlier suggested that the process of receiving and re-paying loans may unfold differently for men and women, with differential impacts on mental health. Further, our previous work in similar populations has revealed important differences along gender lines with respect to adverse selection and moral hazard [45], suggesting that women and men may have different reactions to access to small loans. The existing literature suggests several competing speculations regarding how gender might moderate the association between access to small loans and mental health outcomes. For example, men may benefit more than women from cash loans because they are better able to take advantage of cash alone whereas women may need the support of the group lending mechanism; similarly, in the South African context, men may have more experience engaging in the local economy or may have more societal sway that allows them to take more effective advantage of loan access. Conversely, it is possible that women could benefit more than men because they have fewer outside options for credit and thus may take better advantage of the individual loan option; they could have stronger and more stable social networks to help them make use of a loan, or they could also have greater access to complementary training via NGOs that focus on women.

Our sample composition allowed us to explore these potential differences between women and men in terms of receiving a "second look" for a small individual loan reassessment and mental health outcomes. Because no prior research had directly examined gender differences in the impact of access to individual loans on mental health outcomes, however, there was limited rationale to guide the generation of specific directional hypotheses regarding the moderating influence of participant gender.

## Methods

Our sample frame was comprised of individuals, all from separate households, who had applied to an individual lending organization with branches in Cape Town, Port Elizabeth, and Durban in South Africa. Cape Town is in the Western Cape Province of South Africa and has the second-highest population in the country – after Johannesburg – with over 3.5 million inhabitants. Port Elizabeth is in the Eastern Cape Province and is one of the major seaports of South Africa; it has a population of 1.2 million. Durban, the third largest city in South Africa, is in KwaZulu-Natal in the north-east of the country; its population is less than 3.5 million.

Details on the consumer credit market and the sample collection have been reported previously [46]. Briefly, new applicants to the program were selected between September and November, 2004; these applicants had initially been rejected by the lender but were deemed potentially creditworthy. Six to twelve months later, an independent survey firm assessed 787 of these individuals, with an interview that had been designed by the research team, and included questions on demographics, socio-economic status, subjective social status, major life events, household decision-making, and various indicators of mental health. In some households, the applicant was not present and economic questions were asked of the household head. The mental health questions were only asked when the actual applicant was the respondent. The surveys were conducted in English and translated as needed. Surveyors were able to complete 626 surveys for an 80% response rate. Mental health data were collected in roughly 50% of the cases, producing the final sample size of 257 individuals. A computer programming error on the survey software, intended to randomize the order of questions, instead dropped the mental health questions from half of the sample. Thus, the 50% that received the mental health questions were randomly sampled from the full sample frame of applicants surveyed.

We have previously reported cross-sectional associations between mental health and socio-economic outcomes in this sample [10]. In brief, that paper showed very high levels of both depressive symptoms and perceived stress among participants; based on their depressive symptom scores, 50.4% of men and 64.5% of women exceeded the cut-off at which professional mental healthcare would be recommended in the United States. Additionally, the study found that poorer mental health status was more common among those with lower SES.

Ethics approval for this research – including the element of omission of full disclosure – was obtained from the Princeton University Institutional Review Panel, and the research proposal was reviewed and approved by the legal department of the South African lending organization with whom we worked. Informed consent was obtained from all participants in the study.

### Overview of loan system

The cooperating Lender has operated for over 20 years as one of the largest, most profitable micro-lenders in South Africa, and its product offerings were somewhat differentiated from competitors. Unlike many cash lenders, it did not pursue collection or collateralization strategies such as direct debit from paychecks, or physically keeping bank books and ATM cards of clients. Its pricing was transparent and linear, with no surcharges, application fees, or insurance premiums added to the cost of the loan. In this experiment 98% of the borrowers received the standard loan for first-time borrowers: a 4-month maturity at 11.75% per month, charged on the original balance (200% annual percentage rate). Interest was charged up front (using the "add-on" practice common in consumer loan markets), and the loan was then amortized into 4 equal monthly repayments. Per standard practice in the cash loan market, the Lender conducted underwriting and transactions in its branch network. Its risk assessment technology combined centralized credit scoring with decentralized discretion. The credit scoring model screened out severely unqualified applicants and produced a recommendation on whether to approve the application and then branch personnel made the final decision. The Lender rejected fifty percent of new applications due to unconfirmed employment, suspicion of fraud, poor credit rating, and excessive debt burden.

Applicants who were approved often defaulted on their loan obligation, despite facing several incentives to repay; default rates ranged from 15–20%. Incentives included decreasing prices and increasing future loan sizes following good repayment behavior. Punishment included reporting to credit bureaus, frequent phone calls from collection agents, court summons, and wage garnishments.

### Experimental Design and Operations

**Figure 1:**
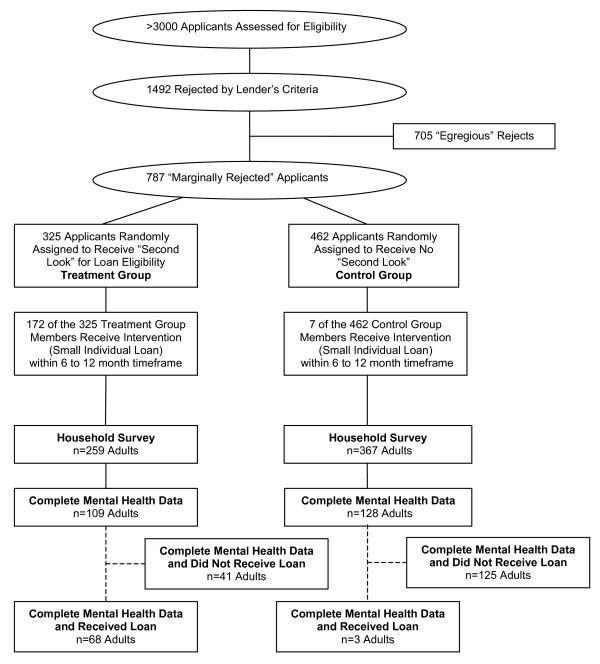
**Sampling framework and randomization**.

The Lender implemented the experiment in a series of steps (Figure 1). First, loan officers evaluated each of the over 3,000 new applicants using the Lender's standard underwriting process and three additional steps. Under normal operations, the loan officer would use a combination of a credit scoring model and her/his own discretion to make a binary approve/reject decision. The experiment forced loan officers to take the first additional step of dividing the "reject" category into two bins. "Marginal" rejects would be eligible for treatment; "egregious" rejects would not be assigned a loan under any circumstances. Egregious rejects were identified subjectively by the officers, based on extremely poor credit history, over-indebtedness, suspected fraud, lack of contactability, or legal problems. Loan officers processed about 1,500 new applications within participating branches during our study period. Seven hundred and five applications were deemed egregious rejects, leaving us with a sample frame of 787 marginally rejected applicants for the experiment. The motivation for experimenting with increases in credit supply on a pool of marginal applicants is twofold. This approach focuses on those who should be targeted by initiatives to expand access to credit, and it also provides the Lender with information about the expected profitability of inducing branch personnel to approve more risky loans.

In the second step of the experiment, randomization software developed for this study was used to encourage loan officers to reconsider randomly selected marginal rejects. The randomization was a simple piece of Windows software that included a data entry screen, where officers inputted client information, and then were presented with a randomization results screen. Random assignment to the Treatment condition constituted being part of a group of applications for which the Lender received "encouragement to reconsider" (i.e. to take a "second look"); those with better credit scores among the marginal rejects were treated with probability 0.50, and those with worse credit scores among the marginal rejects were treated with probability 0.25. The treated group did not receive "randomized approval" for the loan because loan officers had pecuniary incentives to be risk-averse, and the Lender deemed it impractical to force officers to comply strictly with the randomizer's decision. In total, 325 applicants were assigned to receive a "second look," leaving 462 in the Control group. Power calculations had been conducted to determine the relevant sample size necessary to detect differences in employment and the poverty line [46] and were deemed sufficient to test mental health outcome measures; although, as mentioned above, the sample size for the mental health outcomes was inadvertently – albeit randomly – halved.

Finally, the branch manager used his or her discretion to make the final credit decision and announced it to the applicant. Not all who received a second look were approved by the branch manager, and fifty-three percent of the applicants in the Treatment group eventually received a loan; only 2% of applicants in the Control group received a loan during the experimental period. Consistent with commonly-accepted standards for social and economic interventions in which there may be variable "take-up" of the program, we conducted our analysis on a conservative "intent-to-treat" basis [47,48]. Hence we compare those *assigned *to Treatment to those *assigned *to Control, regardless of whether the branch adhered to the random assignment. The applicant was not privy to the loan officer's initial decision, the existence of the software, or the introduction of a randomized step in the decision-making process.

Accepted applicants were offered an interest rate, loan size, and maturity per the Lender's standard underwriting criteria. Loan repayment was monitored and enforced according to normal operations. Branch manager compensation was based in part on loan performance, and the experiment did not change incentive pay.

#### Household data collection

Each survey was conducted within six to twelve months of the date that the applicant entered the experiment by applying for a loan and being placed in the marginal group. In order to avoid potential response bias between the Treatment and Control groups, neither the survey firm nor the respondents were informed about the experiment or any association with the Lender. We told the survey firm that the target households' contact information came from a "consumer database in South Africa."

Our approach minimized three potential sources of bias in the study. First, the timing of the assessment allowed sufficient time for the Control group applicants to find credit elsewhere, reducing the chances of the Treatment group demonstrating benefits purely because of quicker access to credit. Second, the potential impact of the program was evaluated well after the maturity date on the marginal loans. This ensures that we do not simply measure an initial spike of purchasing, and can evaluate the longer term impact of access to the loan. Third, the six to twelve month horizon partially allowed for the fact that some kinds of investments have a gestation period before they manifest in economic outcomes and could be expected to influence participants' mental health. In short, we have chosen to evaluate "medium-run" rather than immediate or long-run impacts.

### Outcome measures

General perceived stress over the past week was assessed using a 10-item version of Cohen's Perceived Stress Scale (PSS) [49]. Sample questions from the scale include: "How often have you been upset because of something that happened unexpectedly?", "How often have you felt that you were unable to control the important things in your life?" and "How often have you been angered because of things that were outside your control?" The participants respond according to a 5-point Likert scale, in which a response of 0 was "not at all" and a response of 4 was "always". Scores range from 0 to 40, and the test had a Cronbach's alpha of 0.72. The test has not to our knowledge been previously used in the African context, but has been used widely in other countries (e.g., Jordan [50], Korea [51], and Spain [52]). Given that there was no prior research to inform us as to a culturally appropriate clinical cut-off in this study population, we used a conservative strategy of setting the 75^th ^percentile as a cut-off to signify "high" stress symptoms.

Depressive symptoms were assessed using the Center for Epidemiologic Studies Depression Scale (CES-D), a 20-item questionnaire designed to assess intensity of depressive symptoms in the past seven days [53,54]. Sample items include: "How often did you feel that everything you did was an effort?", "How often did you feel lonely?", and "How often did you feel sad?" Scores range from 0 to 60, with a generally accepted cut-off score of 16 in the United States for high risk of clinical depression[54]. The CES-D has been used previously in Africa, but not validated [55,56]. In the current study, Cronbach's alpha for the total CES-D score was 0.89. For similar reasons as described above for depressive symptoms, we used a cut-off at the 75^th ^percentile.

### Covariates

Details of the subjects' demographic and socio-economic characteristics were ascertained through questions about gender, age, province (Western Cape, Eastern Cape, or KwaZulu-Natal), educational attainment (dichotomous variable split at grade 12), monthly household income (dichotomous variable split at median), household size, and risk category, which affected the probability of assignment to Treatment. Race was included as African or non-African; the latter category included those who self-identified as white, Indian, and multi-racial.

### Statistical methods

We first examined the differences between the Treatment (those randomly assigned to receive a "second look") and Control (those assigned not to receive a second look) across all socio-demographic variables to ensure that the groups were balanced. We then examined the differences in socio-economic variables within the group assigned to Treatment, comparing those who had actually received a loan and those who had not.

We used tests of proportions to explore in a simple analysis whether Treatment and Control groups were different in terms of primary outcome measures, and then compared within the Treatment group to compare those who had actually received a loan and those who had not; this second analysis has inherent selection bias as it does not adhere to the experimental protocols.

Next, we conducted a multinomial probit regression analysis in which the dependent variables were risk for high depressive symptoms, risk for high perceived stress, and risk for concurrently high depressive symptoms and perceived stress; all of these conditions were in comparison with having low symptoms in both depressive symptoms and perceived stress. The primary independent variable was random assignment to Treatment or Control groups. Beta coefficients and 95% confidence intervals are presented as results.

In the first model, we examined only the treatment effect. In the second model, we further adjusted for age, gender, province, educational attainment, household size and monthly household income. Race was initially included as a covariate, but due to the small proportion of participants that were not of African descent, the cell sizes were unbalanced and estimates of the impact of the randomized intervention, while consistent, were not reliable; thus, race was not included in the multivariate analyses. However, we replicated the multi-variate models just on the subset of people who were of African descent to examine whether race was modifying the results. Given the known differences in prevalence of symptoms of perceived stress and depression by gender, and different social policies targeting females versus males, we then included a Treatment by gender interaction term.

All analyses controlled for month of survey implementation and were adjusted for probability that the participant would receive the loan because better credit scores among the marginal rejects had been treated with probability 0.50, and those with worse credit scores among the marginal rejects were treated with probability 0.25.

As an ancillary analysis, we used whether the participant received the loan as the primary independent variable rather than the intent to treat approach. This comparison did not use the experimental variation provided by the research methodology, and thus should not interpreted as the causal impact, since it also confounds selection biases and reverse causality, as compared to the primary specifications shown in this paper, which do not have such confounds. All statistical analyses were conducted using STATA 9.2 for Windows (STATA Corporation: College Station, TX).

## Results

### Description of sample

Of the 250 participants for whom some mental health data were available, 13 were not included because they didn't have complete scores for both the depressive symptom and perceived stress scales. The sample of participants who had mental health data available (n = 237) were not different from those who did not have the data available (n = 387) across a wide range of socio-demographic variables, although there were some small differences in province of residence (Additional file 1).

Of the final analysis sample of 237, a total of 109 were assigned to Treatment and 128 were assigned to Control. As would be expected given the randomized design of the study, the Treatment and Control groups did not differ significantly on any of the socio-demographic variables (Table 1). The sample was approximately half female, with over two-thirds of the sample of African descent and less than a quarter having received greater than a high school education. The samples were evenly distributed among the three study sites (Eastern Cape, Western Cape and KwaZulu Natal).

**Table 1:** Participant Socio-demographic Characteristics at Baseline, by Treatment Group^1^

	**Assigned to Control** **(n = 128)**	**Assigned to Treatment** **(n = 109)**	**p-value for difference**^2^
**Characteristics**			
Female gender	61 (47.7%)	63 (57.8%)	0.12
Age, years	36.2 (12.0)	35.6 (9.4)	0.76
Education > grade 12	27 (21.2%)	24 (22.0%)	0.89
African Race by self report	84 (65.6%)	79 (73.2%)	0.21
Household size, number	5.4 (3.3)	5.3 (3.2)	0.79
Household monthly income, median (IQR)	1938 (842, 4789)	1979 (1000, 4701)	0.08
Income > sample median	62 (48.4%)	55 (50.5%)	0.76
Province			
Eastern Cape	43 (33.6%)	30 (27.5%)	0.31
Western Cape	39 (30.5%)	46 (42.2%)	0.06
KwaZulu Natal	46 (35.9%)	22 (30.3%)	0.36

^1 ^Treatment was being randomly assigned to receive a second look for a loan application. Mean (SD) or No. (%) presented unless otherwise noted

^2 ^Tests of difference conducted using t-test, test of proportions or non-parametric test of difference between medians where appropriate.

Of the participants assigned to Treatment, there were some significant socio-demographic differences between those who actually received a loan and those who did not (Additional file 2). For instance, those who received the loan were significantly more likely to have received greater than a high school education and to have a higher household income. The groups were not different in terms of gender, age, race, household size or province.

Participants assigned to the Treatment group were more likely to experience high symptoms of stress and low symptoms of depression when compared with those assigned to Control (15.3% versus 6.0%, p = 0.02) (Table 2). There were no other differences when comparing those assigned to Control and those assigned to Treatment in terms of mental health outcomes. Of the study participants who were assigned to Treatment, there were no differences in terms of presence of stress or depression symptoms when comparing those who received the loan and those who did not (Additional file 3).

**Table 2:** Mental Health Symptom^1 ^Distribution (number and percentage) at Follow-up, by Group for Treatment Assignment

**Symptoms**	**Assigned to Control** **(n = 128)**	**Assigned to Treatment** **(n = 109)**	**p-value for difference**^2^
High stress symptoms	29 (22.7%)	34 (31.2%)	0.14
High depression symptoms	37 (28.9%)	26 (23.9%)	0.38
High stress, low depression	8 (6.0%)	17 (15.3%)	0.02
High depression, low stress	16 (12.0%)	9 (8.1%)	0.31
High depression & high stress	21 (15.8%)	17 (15.3%)	0.73
No depression or stress	82 (61.7%)	66 (59.5%)	0.73

^1^High stress symptoms defined as having a score on the Cohen Perceived Stress Scale = 75^th ^percentile; High depressive symptoms defined as having score on the CES-D (Center for Epidemiologic Studies Depression Scale) ≥ 75^th ^percentile.

^2 ^Tests of difference conducted using test of proportions.

### Intent-to-treat impact analysis

The primary impact analysis used multinomial multivariate probit regressions with data for men and women combined (Table 3). Participants who were randomized into receiving a second chance for a loan (Treatment) showed significantly higher levels of perceived stress than those in the intervention group (β = 0.64, 95% CI, 0.04, 1.23), and this finding remained unchanged with the inclusion of covariates (β = 0.78, 95% CI, 0.13, 1.43). Findings were unchanged when the multivariate models were conducted just with the subset of participants that was of African descent. Presence of high stress symptomatology was also positively associated with household size (β = 0.11, 95% CI, 0.01, 0.21).

**Table 3:** Effect of randomized assignment to Treatment to receive a "second look" for a cash loan on having high stress symptoms, high depression symptoms, or both high depression and stress symptoms at Follow-up.^1^

	**High stress symptoms**		**High depression symptoms**		**High depression and stress**	
**Treatment**	0.64* (0.04, 1.23)	0.78* (0.13, 1.43)	**-0.19** (-0.78, 0.40)	**-0.20** (-0.80, 0.41)	**0.03** (-0.50, 0.56)	**-0.02** (-0.57, 0.53)
**Female gender**		**0.06** (-0.57, 0.68)		**0.05** (-0.54, 0.64)		**0.19** (-0.35, 0.73)
**Age, years**		**-0.01** (-0.04, 0.02)		**0.01** (-0.01, 0.03)		**0.00** (-0.03, 0.02)
**Education > 12 years**		**0.25** (-0.53, 1.02)		**-0.33** (-1.21, 0.56)		-1.00* (-1.77, -0.22)
**Household size**		0.11* (0.01, 0.21)		**0.04** (-0.05, 0.13)		**0.07** (-0.01, 0.14)
**Income > median**		**0.28** (-0.36, 0.91)		-0.88* (-1.58, -0.19)		**0.14** (-0.47, 0.74)
**Western Cape**^2^		**-0.51** (-1.25, 0.22)		**-0.34** (-1.05, 0.38)		**-0.53** (-1.24, 0.17)
**KwaZulu Natal**^2^		**-0.67** (-1.40, 0.07)		**-0.05** (-0.80, 0.70)		**0.04** (-0.67, 0.74)

^1 ^* p < 0.05; n = 236 for unadjusted models and n = 235 in adjusted models. Multinomial probit regressions were performed with baseline comparison group as having no stress symptoms and no depressive symptoms. Results using other base categories are presented in the Appendix. Results are presented as beta coefficients with 95% confidence intervals. High stress symptoms defined as having a score on the Cohen Perceived Stress Scale ≥ 75^th ^percentile; High depressive symptoms defined as having score on the CES-D (Center for Epidemiologic Studies Depression Scale) ≥ 75^th ^percentile. Adjusted models also include covariate for month of testing and probability of receiving loan.

^2 ^Eastern Cape is comparison group

Randomization to the Treatment group was not associated with the presence of high depression symptoms, or with the presence of the combination of high depression and high stress symptoms. Having a higher income was associated with having a lower number of symptoms of depression (β = -0.88, 95% CI, -1.58, -0.19); having greater than 12 years of education was significantly associated with having lower combined depression and stress symptoms (β = -1.00, 95% CI, -1.77, -0.22). Results for the primary analysis (using those with low depression/stress symptoms as the comparison group) were virtually identical when using whether the participant actually received the loan as the primary independent variable (data not shown); although, again, it is important to note that this specification confounds selection as it does not conform to the experimental procedure.

**Figure 2:**
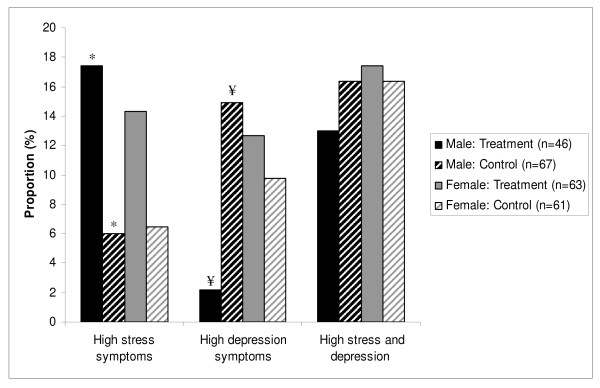
**Symptoms by gender and treatment group^1^**. ^1 ^Tests of differences of proportions between Treatment and Control were conducted separately by gender, with symbols over two columns (*, ¥) indicating that the differences between the two columns with matching symbols are statistically significant (p < 0.05).

The second set of analyses used multinomial probit regressions and included a Treatment by gender interaction term to examine differences by gender (Table 4). Significant Treatment (β = -1.18, 95% CI, -2.34, -0.02) and Treatment by gender interaction terms (β = 1.53, 95% CI, 0.13, 2.93) were evident for high depressive symptoms; both coefficients were significant with the inclusion of covariates. This interaction indicated that being randomized to the Treatment condition was associated with significantly lower level of depressive symptoms for men, but not women (Figure 2). None of the other Treatment by gender interaction terms was significant.

**Table 4:** Effect of randomized assignment to Treatment to receive a "second look" for a cash loan on having high stress symptoms, high depression symptoms, or both high depression and stress symptoms with treatment*gender interaction at Follow-up.^1^

	**High stress symptoms**		**High depression symptoms**		**High depression and stress**	
**Treatment**	**0.59** (-0.27, 1.46)	**0.65** (-0.26, 1.57)	-1.18* (-2.34, -0.02)	-1.32* (-2.56, -0.07)	**-0.23** (-1.03, 0.57)	**-0.36** (-1.18, 0.46)
**Female gender**	**-0.02** (-0.92, 0.89)	**-0.02** (-0.98, 0.94)	**-0.34** (-1.11, 0.43)	**-0.65** (-1.44, 0.13)	**-0.07** (-0.79, 0.65)	**-0.09** (-0.82, 0.63)
**Treatment × Female**	**0.08** (-1.12, 1.28)	**0.23** (-1.09, 1.54)	1.53* (0.13, 2.93)	1.87* (0.37, 3.38)	**0.46** (-0.62, 1.54)	**0.63** (-0.48, 1.74)
**Age, years**		**-0.13** (-0.04, 0.02)		**0.01** (-0.02, 0.03)		**0.00** (-0.03, 0.02)
**Education > 12 years**		**0.22** (-0.56, 1.00)		**-0.44** (-1.36, 0.49)		-1.05* (-1.82, -0.28)
**Household size**		0.11* (0.01, 0.21)		**0.05** (-0.04, 0.14)		**0.07** (-0.01, 0.15)
**Income > median**		**0.27** (-0.37, 0.91)		-0.95* (-1.66, -0.25)		**0.11** (-0.49, 0.72)
**Western Cape**^2^		**-0.51** (-1.24, 0.22)		**-0.24** (-0.95, 0.47)		**-0.52** (-1.23, 0.18)
**KwaZulu Natal**^2^		**-0.69** (-1.43. 0.06)		**-0.08** (-0.85, 0.68)		**0.02** (-0.68, 0.73)

^1 ^* p < 0.05; n = 236 for unadjusted models and n = 235 in adjusted models. Multinomial probit regressions were performed with baseline comparison group as having no stress symptoms and no depressive symptoms. Results are presented as beta coefficients with 95% confidence intervals. High stress symptoms defined as having a score on the Cohen Perceived Stress Scale ≥ 75^th ^percentile; High depressive symptoms defined as having score on the CES-D (Center for Epidemiologic Studies Depression Scale) ≥ 75^th ^percentile. Adjusted models also include covariate for month of testing and probability of receiving loan.

^2 ^Eastern Cape is comparison group

## Discussion

The data presented here suggest that randomization into receiving a second chance for a small individual loan is associated with increased levels of perceived stress among the general sample, with important differences by gender. Among men randomly allocated to receive a second chance for a cash loan, there were increased symptoms of perceived stress and decreased depressive symptoms. Among women, however, the findings were not significant. This paper is among the first to demonstrate empirically with a randomized design that participation in a program to provide greater access to small loans has mixed effects on mental health.

Very few studies have been able to examine how SES and mental health are causally linked and most have relied on cross-sectional analyses. Although there are several methodological limitations to the study reported here that will be discussed below, our study was designed to test the primary hypothesis that an improvement in SES brought about through increased access to a small, individual loan could improve mental health outcomes; we did indeed find reductions in depression in men. This hypothesis was supported by many cross-sectional studies examining the associations between low SES and increased risk for mental health problems [12], but was also supported in part by findings from a seven year longitudinal study conducted in Belgium showing that decreases in SES as measured by material standard of living were related to later increases in prevalence of depressive symptoms [57]. A similar cross-sectional study in Indonesia examined differences in rates of development and also found that increases in economic development were associated with fewer psychological symptoms [58]. In spite of the fact that most of the literature discusses SES as the factor determining mental health status, we cannot ignore the possibility that the presence of mental health symptoms could itself be a risk factor for low SES.

This study contributes uniquely to the literature by examining critical mental health outcomes and how they are related to participation in a small loan program designed to improve SES in a low-income population in South Africa. A clear problem in comparing our study to previous studies that have looked at SES and mental health is that there are a large number of ways to measure SES, there are methodological differences in outcome measures, heterogeneous samples and differences across countries in terms of degree of economic development. Among women in Bangladesh, for example, those living in poverty – defined as living in a household with a small amount of land and having at least one household member selling manual labor for survival – and those with lower education were more likely to report emotional distress when compared with those not living in poverty [41]. Within the sample of women living in poverty, however, those who were perceived to be contributing to household income through participation in the labor market – which could be perceived as *increasing *their household SES – reported more emotional stress than those who were not contributing to household income. Similarly, in a cross-sectional study in Brazil, participation in the informal labor market – e.g. street vending, domestic jobs – was associated with higher scores on a psychiatric symptoms questionnaire [59]. A cross-sectional study in India showed that increased mental distress was associated both with being employed and with having smaller rather than larger landholdings [40]. Although it is seemingly contradictory that depressive symptoms or mental distress could be linked both to markers of higher and lower SES, previous research has shown that methods for measuring SES operate differently according to context and vary greatly in the way that they relate to health outcomes [60].

Our findings indicate that men in the Treatment group exhibited higher perceived stress but lower levels of depressive symptoms when compared with men in the Control group and these results suggest that the impact of "taking up" a small individual loan may differ by gender. The reduction in depressive symptoms fit with our hypotheses, but the reverse pattern shown for perceived stress among men is intriguing. While counterintuitive in the sense that high levels of perceived stress are often *positively *associated with depressive symptoms, there is also a body of research that demonstrates that even "good" major life events such as graduations, new jobs, or marriages can be experienced as stressful for some [61]. Thus, it is possible that the men in the Treatment group experienced increases in perceived stress as they took up the loans and engaged in new economic activities which ultimately demonstrated longer-term benefits for their mental health. Other data collected in the same study suggests that for those participants who were randomized into the Treatment group, there were significant and positive effects on job retention, income, food consumption quality and quantity, and household decision-making control [46], all of which could explain the reduction in depressive symptoms seen in men.

A handful of small, qualitative studies support the notion that micro-loans to the poor – both in the form of small individual loans to a bank or more organized microcredit schemes – may be triggers for increased stress [38,41]. With increased access to credit, people who take out loans may be forced to shoulder a dual burden in which they work both inside and outside the home [62]. In some cases, people in the household may be forced to take out loans by family members, and then if the income is scarce, the person who took out the loan is burdened with the debt [63]. It may also be that some people living in poverty do not want to operate as entrepreneurs and yet they may feel forced to participate in an entrepreneurial venture because it is their only option [64]. Prior non-experimental research in an integrated, group-model microcredit scheme in Bangladesh has suggested that women in highly patriarchal societies who participate in microcredit may experience distress stemming from newly adopted nontraditional gender roles, as well as the disappointment of unmet expectations if no real economic benefits are experienced [41]. While the present study makes several distinct contributions to the field in its use of randomized assignment of participants to receive small individual loans and the assessment of perceived stress and depression using well-established measures, we do not have data that specify the kinds of benefits or stressors that individuals experienced as a result of their participation or non-participation in the microcredit loan program. That is, we did not directly study the pathways by which randomization to the experimental condition may have influenced levels of perceived stress and depressive symptoms; this important question should be explored in future research.

The primary limitations to interpreting and generalizing from this study are as follows. First, as is true of any society, circumstances in the South African credit market may be fundamentally different than other societies, and thus replication of this study is necessary in other contexts both within and outside of South Africa. Second, the sample frame of this study was the "marginal" clients of the Lender, not the full sample of those who borrow in this market, and thus may not be representative of all borrowers. From a policy perspective, however, this may be the population of most interest, since interventions aimed at encouraging lenders to expand access to credit can be expected to expand access exactly at that margin to those currently being rejected but seeking loans. Third, this credit market and terms of credit are substantially different than other models of microfinance. There is no focus on entrepreneurial credit as many microfinance organizations have, such as the Grameen Bank in Bangladesh; there is no group aspect to the lending process (which may ameliorate or exacerbate changes in mental health outcome); and, the interest rates are considerably higher than those offered by many NGOs in other developing countries (200% annualized percentage rate). All of these factors imply that our findings relating to small individualized cash loans cannot be extended to understanding the potential impact of other loan schemes. Fourth, we do not have baseline information on the mental health outcome measures and only have information from follow-up. However, the groups were balanced across several socio-economic variables at baseline, which suggests that they were likely to have been balanced according to mental health measures as well. Fifth, we do not know how the loans were used (i.e., for new business generation of consumption) and thus are limited in our ability to discuss mechanisms. Finally, the survey instruments have not been previously validated in South Africa, so they may not accurately capture local conceptions or manifestations of mental illness.

## Conclusion

Psychological health is becoming an increasingly important component of the concept of health in the developing world [65]. Our findings suggest that one mechanism used to reduce the economic stress of extremely poor individuals can have mixed effects on their levels of psychological stress and symptomatology. Our data support the notion that mental health should be included as a measure of success (or failure) when examining potential tools for poverty alleviation. Further longitudinal research is needed to replicate the findings of the present study, and future research should use quantitative and qualitative methods to examine how small individual loans affect the roles, daily activities, and concerns of individual and families to help explain the linkages between access to loans and mental health outcomes. The differential effects for men and women found here should also be investigated further. Such research should ideally include assessments of economic resources and psychological functioning at baseline and at multiple points during and after the loan period if feasible.

## Competing interests

The authors declare that they have no competing interests.

## Authors' contributions

LCHF contributed to the study's concept and design, conducted statistical analyses and drafted the manuscript. RH contributed to the statistical analysis for the study and wrote portions of the manuscript. EO wrote portions of the manuscript. DK and JZ conceptualized and designed the study, obtained funding, acquired data and wrote portions of the manuscript. All authors provided critical revision of the manuscript for important intellectual content, read and approved the final manuscript.

## Pre-publication history

The pre-publication history for this paper can be accessed here:



## Supplementary Material

Additional file 1 **Supplemental Table 1**. Socio-demographic Characteristics at Baseline, by Availability of Mental Health data^1^. Click here for file

Additional file 2 **Supplemental table 2**. Participant Socio-demographic Characteristics at Baseline within Treatment group, split by whether received loan or not. Click here for file

Additional file 3 **Supplemental Table 3**. Mental Health Symptom Distribution at Follow-up within those Assigned to Treatment split by whether received loan or not. Click here for file
