# Supplementary material for: Small individual loans and mental health: a randomized controlled trial among South African adults
Source: BMC Public Health. 2008 Dec 16;8:409. doi: 10.1186/1471-2458-8-409 (PMC2647927; doi:10.1186/1471-2458-8-409)
Supplement: Additional file 1 — Supplemental Table 1. Socio-demographic Characteristics at Baseline, by Availability of Mental Health data1. [file 1471-2458-8-409-S1.doc]

**Supplemental Table 1:** Socio-demographic Characteristics at Baseline, by Availability of Mental Health data^1^

|  | **Mental Health Data Available**  **(n=237)** | **Mental Health Data Not Available**  **(n=387)** | **p-value for difference**^2^ |
| --- | --- | --- | --- |
| **Characteristics** |  |  |  |
| Female gender | 124 (52.3%) | 261 (47.4%) | 0.21 |
| Age, years | 35.4 (10.8) | 35.2 (11.4) | 0.81 |
| Education > grade 12 | 51 (21.6%) | 71 (18.9%) | 0.42 |
| African Race by self report | 163 (69.1%) | 348 (63.4%) | 0.13 |
| Household size, number | 5.4 (3.3) | 5.4 (3.0) | 0.94 |
| Household monthly income, median (IQR) | 1979 (900, 4735) | 2233 (1026, 4574) | 0.21 |
| Income > sample median | 117 (49.4%) | 197 (50.64%) | 0.76 |
| Province |  |  |  |
| Eastern Cape | 73 (30.80%) | 123 (31.6%) | 0.83 |
| Western Cape | 85 (35.9%) | 172 (44.2%) | 0.04 |
| KwaZulu Natal | 79 (33.3%) | 94 (24.2%) | 0.01 |

^1^ Mean (SD) or No. (%) presented unless otherwise noted

^2^ Tests of difference conducted using t-test, test of proportions or non-parametric test of difference between medians where appropriate.
