# Supplementary material for: Small individual loans and mental health: a randomized controlled trial among South African adults
Source: BMC Public Health. 2008 Dec 16;8:409. doi: 10.1186/1471-2458-8-409 (PMC2647927; doi:10.1186/1471-2458-8-409)
Supplement: Additional file 2 — Supplemental table 2. Participant Socio-demographic Characteristics at Baseline within Treatment group, split by whether received loan or not. [file 1471-2458-8-409-S2.doc]

**Supplemental Table 2:** Participant Socio-demographic Characteristics at Baseline within Treatment group, split by whether received loan or not^1^

|  | **Assigned to Treatment** | |  |
| --- | --- | --- | --- |
|  | **Received Loan**  **(n=68)** | **Did Not Receive Loan**  **(n=41)** | **p-value for difference**^2^ |
| **Characteristics** |  |  |  |
| Female gender | 40 (58.8%) | 23 (56.1%) | 0.78 |
| Age, years | 35.2 (9.3) | 36.3 (9.6) | 0.57 |
| Education > grade 12 | 19 (27.9%) | 5 (12.2%) | 0.05 |
| African Race by self report | 45 (67.2%) | 34 (82.9%) | 0.07 |
| Household size, number | 5.2 (3.1) | 5.5 (3.6) | 0.71 |
| Household income, median (IQR) | 3042 (1300, 6105) | 1279 (502, 2232) | 0.02 |
| Income > sample median | 43 (63.2%) | 12 (29.3%) | <0.001 |
| Province |  |  |  |
| Eastern Cape | 15 (23.5%) | 13 (34.2%) | 0.23 |
| Western Cape | 28 (41.2%) | 18 (43.9%) | 0.78 |
| KwaZulu Natal | 24 (35.3%) | 9 (22.0%) | 0.14 |

^1^ Treatment was being randomly assigned to received a second look for a loan application. Mean (SD) or No. (%) presented unless otherwise noted

^2^ Tests of difference conducted using t-test, test of proportions or non-parametric test of difference between medians where appropriate.
