# Supplementary material for: Small individual loans and mental health: a randomized controlled trial among South African adults
Source: BMC Public Health. 2008 Dec 16;8:409. doi: 10.1186/1471-2458-8-409 (PMC2647927; doi:10.1186/1471-2458-8-409)
Supplement: Additional file 3 — Supplemental Table 3. Mental Health Symptom Distribution at Follow-up within those Assigned to Treatment split by whether received loan or not. [file 1471-2458-8-409-S3.doc]

**Supplemental Table 3:** Mental Health Symptom Distribution at Follow-up within those Assigned to Treatment split by whether received loan or not^1^

|  | **Assigned to Treatment** | |  |
| --- | --- | --- | --- |
| **Symptoms** | **Received Loan**  **(n=68)** | **Did Not Receive Loan**  **(n=41)** | **p-value for difference**^2^ |
| High stress, low depression | 12 (17.7)% | 5 (12.2%) | 0.45 |
| High depression, low stress | 4 (5.9%) | 5 (12.2%) | 0.25 |
| High depression & high stress | 10 (14.7%) | 7 (17.1%) | 0.74 |
| No depression or stress | 42 (61.8%) | 24 (58.5%) | 0.74 |

^1^High stress symptoms defined as having a score on the Cohen Perceived Stress Scale ≥75^th^ percentile; High depressive symptoms defined as having score on the CES-D (Center for Epidemiologic Studies Depression Scale) ≥75^th^ percentile.

^2^ Tests of difference conducted using test of proportions
